# Supplementary material for: Growth capacity and mortality burden shape microbial persistence under osmotic stress in Bacteroides thetaiotaomicron
Source: bioRxiv. 2026 Sep 9:2026.09.08.750096. Preprint. [Version 1] doi: 10.64898/2026.09.08.750096 (PMC13622673; doi:10.64898/2026.09.08.750096)
Supplement: Supplementary file 1 [file NIHPP2026.09.08.750096V1-supplement-1.pdf]

bioRxiv preprint doi: https://doi.org/10.64898/2026.09.08.750096; this version posted September 9, 2026. The copyright holder for this preprint (which was not certified by peer review) is the author/funder, who has granted bioRxiv a license to display the preprint in perpetuity. It is made available under a CC-BY 4.0 International license.

SUPPLEMENTAL FIGURES

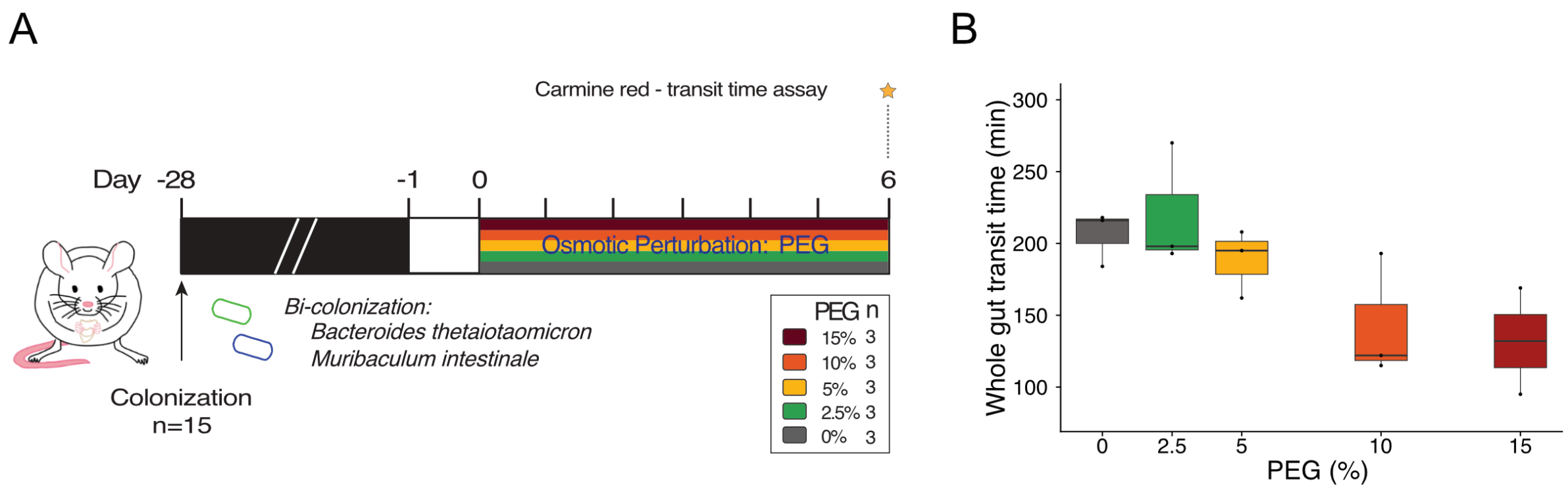

FIGURE S1: Whole gut transit time decreases following PEG treatment. (A) Experimental schematic and sample collection protocol. Germ-free mice (n=15) were colonised with *B. thetaiotaomicron* and *M. intestinale* and allowed to equilibrate for 28 days. Mice were treated with 0%, 2.5%, 5%, 10%, 15% PEG in drinking water (shown by color) *ad libitum* for 6 days after which they were gavaged with carmine red and monitored for dye in the stool to measure transit time. **(B)** Whole gut transit time measured after 6 days of treatments with 5 distinct PEG dosages n=3 each).

bioRxiv preprint doi: https://doi.org/10.64898/2026.09.08.750096; this version posted September 9, 2026. The copyright holder for this preprint (which was not certified by peer review) is the author/funder, who has granted bioRxiv a license to display the preprint in perpetuity. It is made available under a CC-BY 4.0 International license.

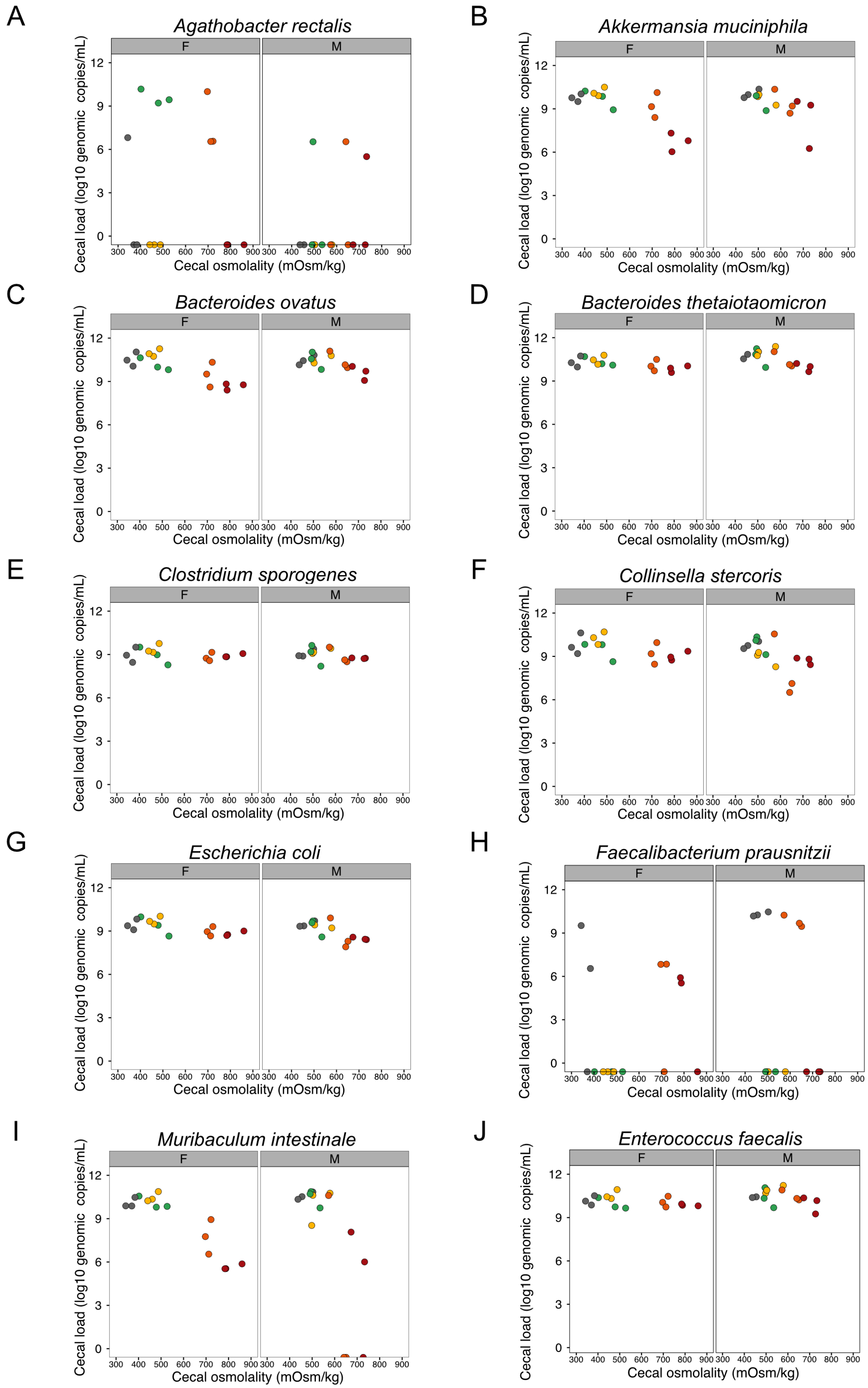

bioRxiv preprint doi: https://doi.org/10.64898/2026.09.08.750096; this version posted September 9, 2026. The copyright holder for this preprint (which was not certified by peer review) is the author/funder, who has granted bioRxiv a license to display the preprint in perpetuity. It is made available under a CC-BY 4.0 International license.

FIGURE S2: Species-specific absolute quantification reveals distinct osmotolerance levels in a defined 10-member community. Absolute abundance (log_{10} genomic copies/mL) measured by species-specific droplet digital PCR (ddPCR) from cecal samples collected after 6 days of PEG treatment (see Fig. 1A for experimental schematic). Individual panels display data for each community member: **(A)** *Agathobacter rectalis*, **(B)** *Akkermansia muciniphila*, **(C)** *Bacteroides ovatus*, **(D)** *Bacteroides thetaiotaomicron*, **(E)** *Clostridium sporogenes*, **(F)** *Collinsella stercoris*, **(G)** *Escherichia coli*, **(H)** *Faecalibacterium prausnitzii*, **(I)** *Muribaculum intestinale*, and **(J)** *Enterococcus faecalis*. Individual points represent individual mice plotted against their respective cecal osmolality (mOsm/kg). Plots are faceted by the sex of the mice (F, female; M, male). Points are color-coded by PEG dosage (0%, 2.5%, 5%, 10%, and 15% PEG) according to the color scheme in Fig. 1A.

bioRxiv preprint doi: https://doi.org/10.64898/2026.09.08.750096; this version posted September 9, 2026. The copyright holder for this preprint (which was not certified by peer review) is the author/funder, who has granted bioRxiv a license to display the preprint in perpetuity. It is made available under a CC-BY 4.0 International license.

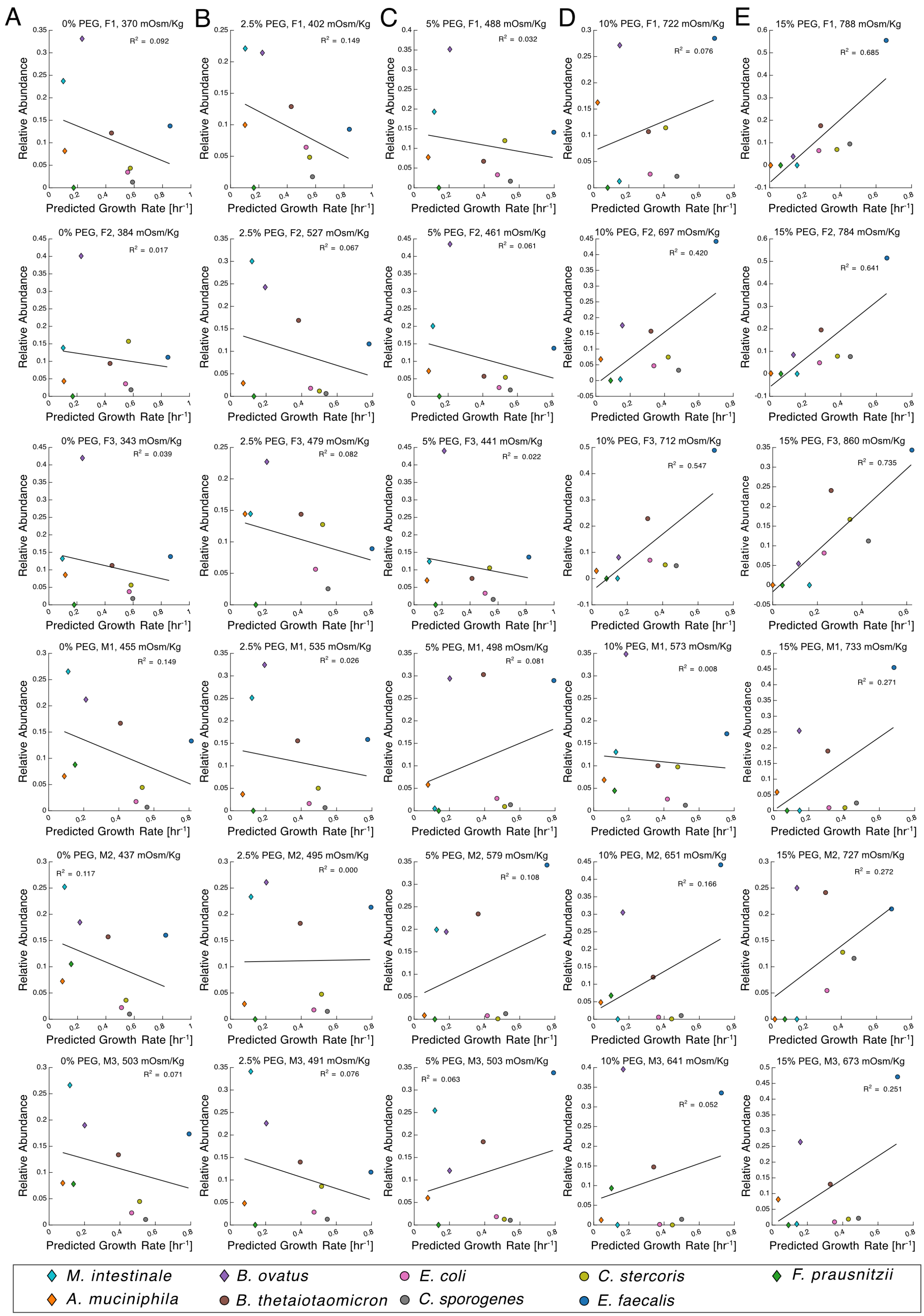

bioRxiv preprint doi: https://doi.org/10.64898/2026.09.08.750096; this version posted September 9, 2026. The copyright holder for this preprint (which was not certified by peer review) is the author/funder, who has granted bioRxiv a license to display the preprint in perpetuity. It is made available under a CC-BY 4.0 International license.

FIGURE S3: Mouse-specific linear regressions between *in vivo* relative abundance and predicted *in vitro* growth rate. Columns represent the experimental PEG treatment groups in the experiment outlined in Fig. 1A: (**A**) 0% PEG, (**B**) 2.5% PEG, (**C**) 5% PEG, (**D**) 10% PEG, and (**E**) 15% PEG. Rows represent individual mice, labeled by sex and numerical identifier (F1-F3 for females; M1-M3 for males) along with their measured cecal osmolality (mOsm/kg) at endpoint. Within each panel, the *in vivo* relative abundance of the 10 community members is plotted against their predicted *in vitro* growth rate at that mouse's specific cecal osmolality. Individual points represent distinct bacterial strains, color-coded by species according to the legend at the bottom. The individual Pearson correlation coefficients derived from these 30 independent linear regressions were compiled to generate the summary correlation profile shown in Fig. 1G.

bioRxiv preprint doi: https://doi.org/10.64898/2026.09.08.750096; this version posted September 9, 2026. The copyright holder for this preprint (which was not certified by peer review) is the author/funder, who has granted bioRxiv a license to display the preprint in perpetuity. It is made available under a CC-BY 4.0 International license.

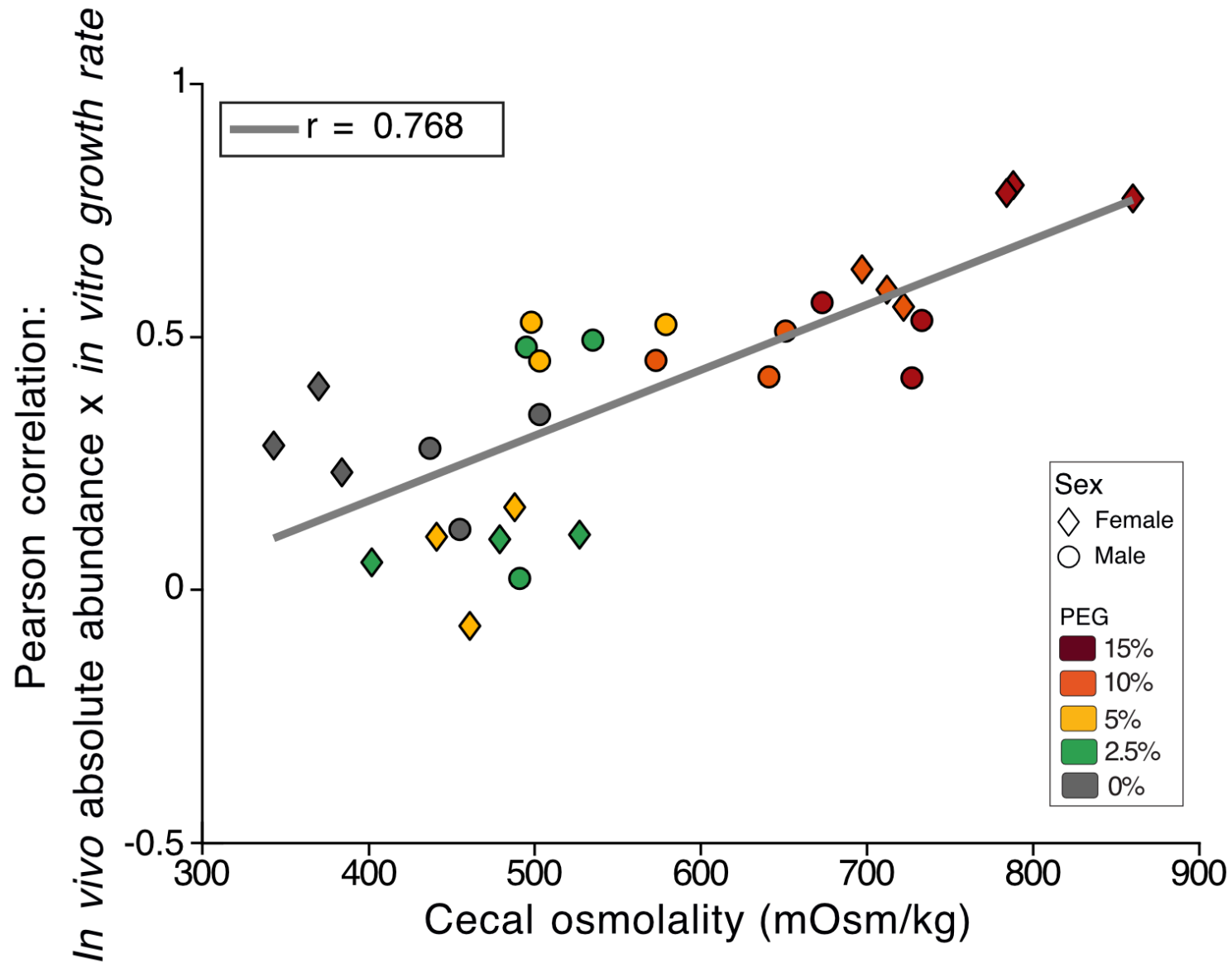

FIGURE S4: Pearson correlation between predicted *in vitro* growth rate and *in vivo* absolute abundance across cecal osmolalities. Pearson correlation coefficients calculated from linear regressions between species-specific *in vivo* absolute abundance measured via ddPCR (see Fig. S2) and predicted *in vitro* maximum growth rates. Individual points represent individual mice plotted against their measured cecal osmolality. Color denotes PEG dosage according to the scheme in Fig. 1A, and shape represents the sex of the mice (triangles for females, circles for males).

bioRxiv preprint doi: https://doi.org/10.64898/2026.09.08.750096; this version posted September 9, 2026. The copyright holder for this preprint (which was not certified by peer review) is the author/funder, who has granted bioRxiv a license to display the preprint in perpetuity. It is made available under a CC-BY 4.0 International license.

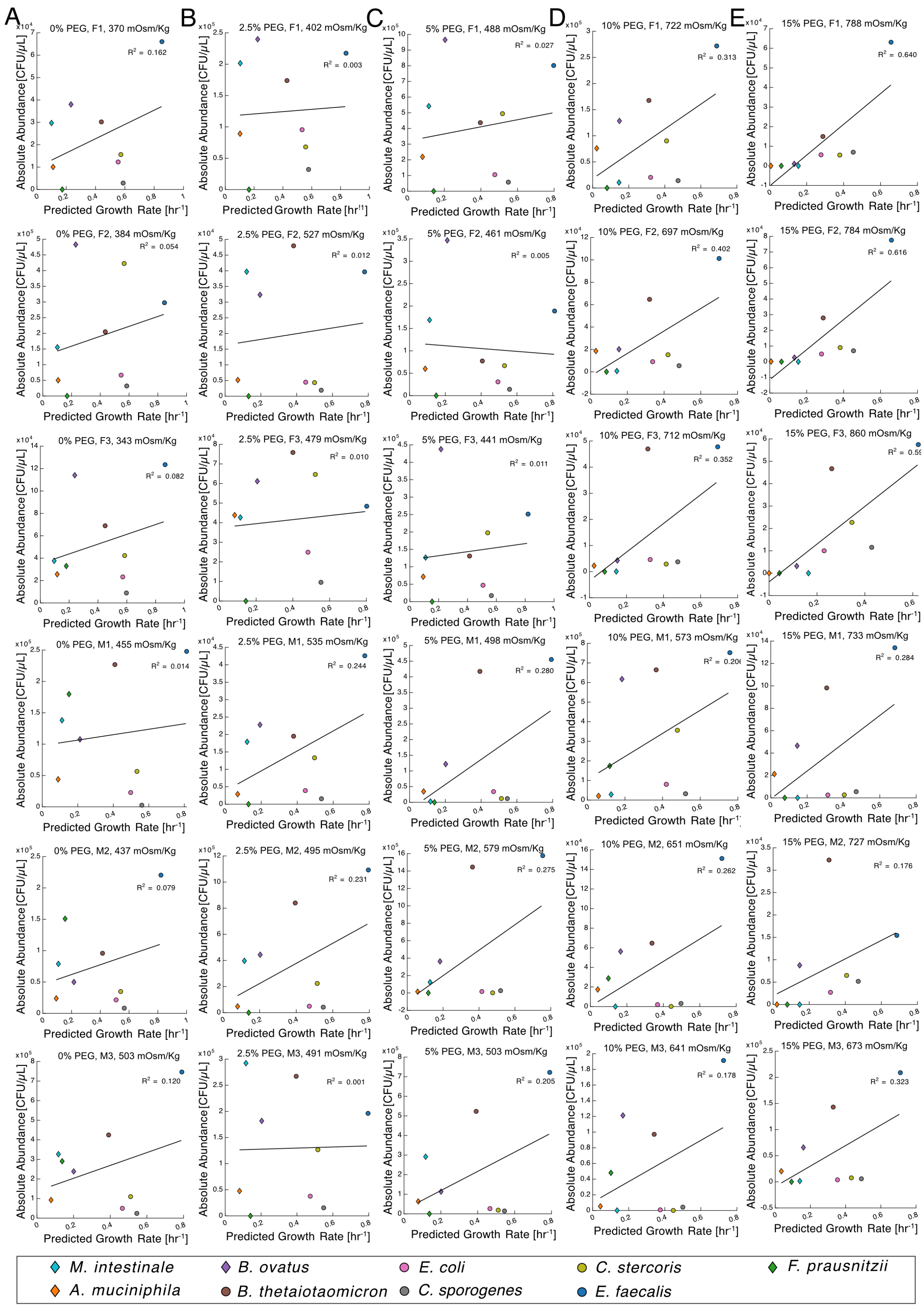

bioRxiv preprint doi: https://doi.org/10.64898/2026.09.08.750096; this version posted September 9, 2026. The copyright holder for this preprint (which was not certified by peer review) is the author/funder, who has granted bioRxiv a license to display the preprint in perpetuity. It is made available under a CC-BY 4.0 International license.

FIGURE S5: Mouse-specific linear regressions between *in vivo* absolute abundance and predicted *in vitro* growth rate. Columns represent the experimental PEG treatment groups in the experiment outlined in Fig. 1A: (**A**) 0% PEG, (**B**) 2.5% PEG, (**C**) 5% PEG, (**D**) 10% PEG, and (**E**) 15% PEG. Rows represent individual mice, labeled by sex and numerical identifier (F1-F3 for females; M1-M3 for males) along with their measured cecal osmolality (mOsm/kg) at endpoint. Within each panel, the *in vivo* absolute abundance of the 10 community members is plotted against their predicted *in vitro* growth rate (hr^{-1}) at that mouse's specific cecal osmolality. Individual points represent distinct bacterial strains, color-coded by species according to the legend at the bottom. The individual Pearson correlation coefficients derived from these 30 independent linear regressions were compiled to generate the summary correlation profile shown in Fig. S4.

bioRxiv preprint doi: https://doi.org/10.64898/2026.09.08.750096; this version posted September 9, 2026. The copyright holder for this preprint (which was not certified by peer review) is the author/funder, who has granted bioRxiv a license to display the preprint in perpetuity. It is made available under a CC-BY 4.0 International license.

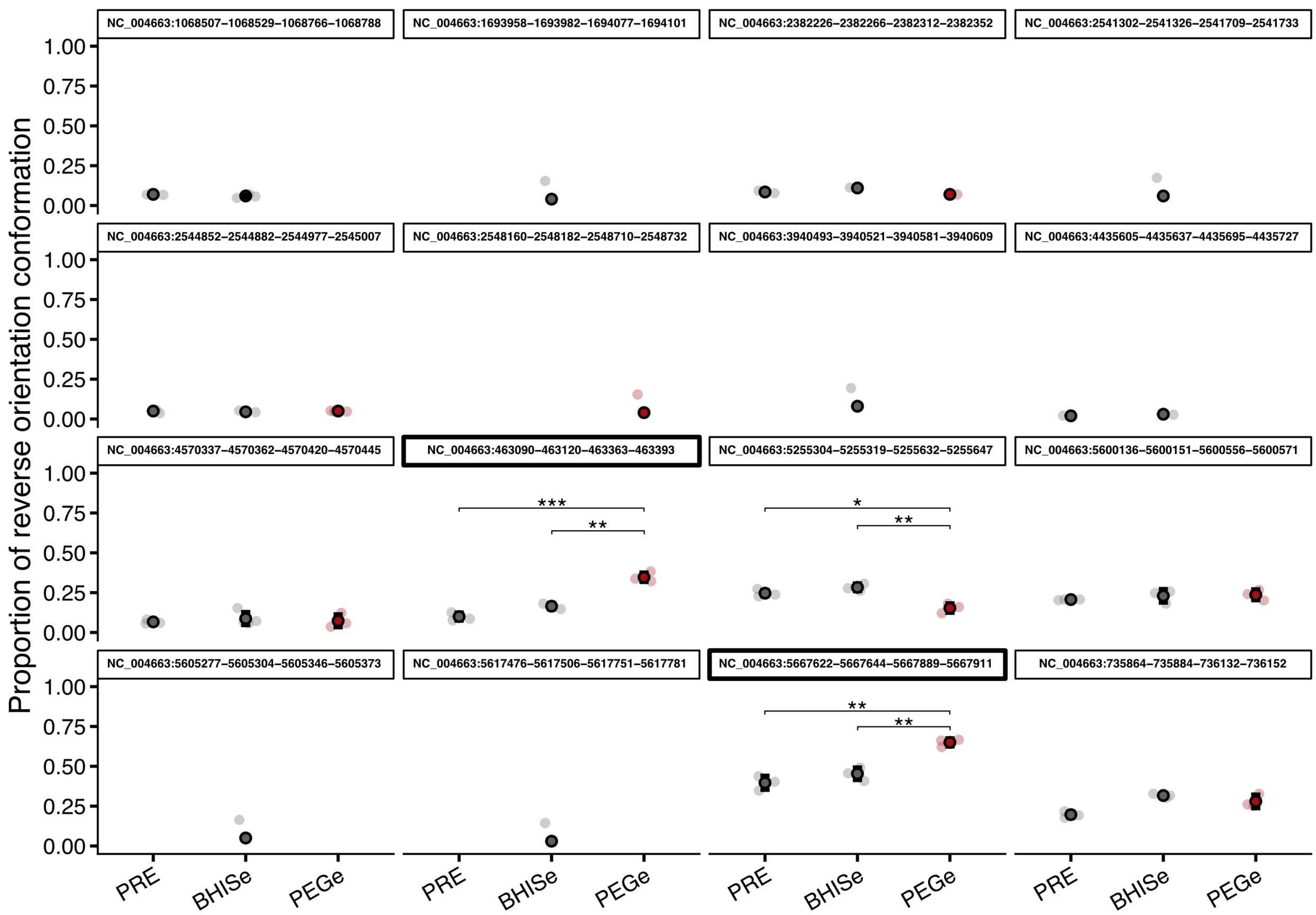

FIGURE S6: PhaseFinder screening of invertible promoters across *B. thetaiotaomicron* populations. Proportion of reverse promoter conformations across all candidate phase variants identified via PhaseFinder after the first passaging round. Only invertons with a minimum of 20 reads mapping to the reverse conformation in at least one population are displayed. Faint points represent individual biological replicates (n=3), bold points indicate the mean, and error bars denote the standard deviation. Panels outlined with thick black borders highlight the only two invertons that exhibited a significant increase in reverse conformation frequency in PEGe populations compared to controls: near the CPS1 cluster (third row, second column) and the PUL78/80 operon (fourth row, third column). Statistical significance was determined by a two-tailed, paired t-test (*p < 0.05, **p < 0.01, ***p < 0.001).

bioRxiv preprint doi: https://doi.org/10.64898/2026.09.08.750096; this version posted September 9, 2026. The copyright holder for this preprint (which was not certified by peer review) is the author/funder, who has granted bioRxiv a license to display the preprint in perpetuity. It is made available under a CC-BY 4.0 International license.

SUPPLEMENTAL TABLES

TABLE S1: Oligos used in this study.

TABLE S2: Raw breseq output files from shotgun sequencing of passaged *B. thetaiotaomicron* populations, related to Figure 2.

TABLE S3: Genetic parts and primers used in this study to engineer wild type *B. thetaiotaomicron*, related to Figure 3.

TABLE S4: Strains and plasmids used in this study to engineer wild type *B. thetaiotaomicron*, related to Figure 3.
